# Successful Haploidentical Hematopoietic Stem Cell Transplantation for Chemotherapy‐Induced Aplastic Anemia in a Breast Cancer Survivor: A Case Report and Literature Review

**DOI:** 10.1155/crh/1212359

**Published:** 2026-07-08

**Authors:** Xin-Yi Qin, Yan-Fang Zhang, Ying Zhang, Yun Luo

**Affiliations:** ^1^ Hematology, The Second Affiliated Hospital of Chongqing Medical University, Chongqing, China, cqmu.edu.cn

**Keywords:** aplastic anemia, breast cancer, chemotherapy, haploidentical hematopoietic stem cell transplantation, myelosuppression

## Abstract

Chemotherapy‐induced aplastic anemia (AA) is a rare but potentially life‐threatening complication in solid tumor treatment. While allogeneic hematopoietic stem cell transplantation (HSCT) is a recognized treatment for AA, haploidentical HSCT (haplo‐HSCT) for chemotherapy‐induced AA in solid tumor patient survivors remains largely unexplored. We report a unique case of successful haplo‐HSCT in a breast cancer survivor who developed AA following chemotherapy. In addition, we conducted a comprehensive literature review on chemotherapy‐induced AA in breast cancer patients and its management, with a focus on HSCT outcomes. This case demonstrates the potential efficacy of haplo‐HSCT in treating chemotherapy‐induced AA in breast cancer survivors. It highlights an important therapeutic option for this rare but severe complication, particularly when matched donors are unavailable. Our experience contributes valuable insights to the limited literature on managing this challenging clinical scenario.

## 1. Introduction

Breast cancer remains one of the most prevalent malignancies among women, with incidence rates continuing to rise globally [1]. The standard treatment approach often combines surgery with chemotherapy. While chemotherapeutic agents effectively target tumor cells, they also indiscriminately damage normal hematopoietic and immune cells, potentially leading to cellular gene rearrangements and mutations. This phenomenon, known as hematologic toxicity, affects approximately 20%–40% of patients [2]. The majority of patients with myelosuppression after chemotherapy can recover their blood cell counts with hematopoietic stimulating factors. However, prolonged hematopoietic dysfunction poses significant risks, including infection and bleeding. Aplastic anemia (AA), a hematologic disorder characterized by bone marrow failure, can arise from various factors, including drug exposure, viral infections, ionizing radiation, immune dysregulation, and genetic predisposition. The primary manifestations of AA include anemia, bleeding tendency, and susceptibility to infections. Current treatment modalities, such as immunosuppressive therapy (IST) and hematopoietic stem cell transplantation (HSCT), have significantly improved patient survival rates and prognoses. We report a case of a breast cancer patient who developed AA following chemotherapy. The patient underwent haploidentical HSCT (haplo‐HSCT), which successfully restored hematopoiesis.

## 2. Case Report

A 46‐year‐old woman presented to our hospital’s outpatient clinic on June 16, 2022, for a breast ultrasound. The examination revealed a 26 × 20 × 20 mm hypoechoic nodule in the left breast, 30 mm from the nipple at the 2 o’clock position (BI‐RADS Category 4c). Additional findings included dilated ducts in the left breast (BI‐RADS Category 2) and no locoregional lesions in the right breast (BI‐RADS Category 1). A left breast biopsy performed on June 17, 2022, confirmed invasive carcinoma with ER (+) 80%, PR (+) 80%, Her‐2 (3+), and Ki‐67 (+) 60%. After excluding surgical contraindications, the patient underwent unilateral breast‐conserving modified radical mastectomy with sentinel lymph node biopsy and fascioplasty on June 23, 2022. Postoperative pathology revealed invasive carcinoma of the left breast (nonspecialized type, NGS Grade II, 6 points) without neural invasion or involvement of surgical margins. Sentinel lymph nodes (0/4) were negative for metastasis. Immunohistochemistry showed ER (90% moderate +), PR (90% strong +), Her‐2 (3+), and Ki‐67 (+) 30%. The patient received six cycles of neoadjuvant chemotherapy (TCbHP regimen: albumin paclitaxel, carboplatin, pertuzumab, and trastuzumab) between July 7, 2022, and October 22, 2022. This was followed by seven cycles of targeted therapy (pertuzumab and trastuzumab) from November 12, 2022, to March 20, 2023. On March 20, 2023, blood tests showed the following: white blood cell count 11.28 × 10^9^/L, absolute neutrophil count 10.17 × 10^9^/L, and platelet count 69 × 10^9^/L. Platelet count improved after supportive treatment.

Postdischarge, the patient reported fatigue and developed skin petechiae without hematemesis or melena. A follow‐up blood test on April 8, 2023, at a local hospital showed: leukocyte count 3.75 × 10^9^/L, erythrocyte count 3.33 × 10^12^/L, hemoglobin 108 g/L, and platelet count 17 × 10^9^/L. Despite treatment with platelet transfusion, interleukin‐11, and other platelet‐elevating therapies for suspected postchemotherapy myelosuppression, the patient’s condition did not improve.

**FIGURE 1:**
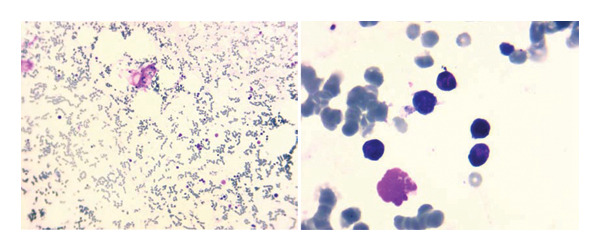
Images of the patient’s bone marrow examination.

**Figure 2:**
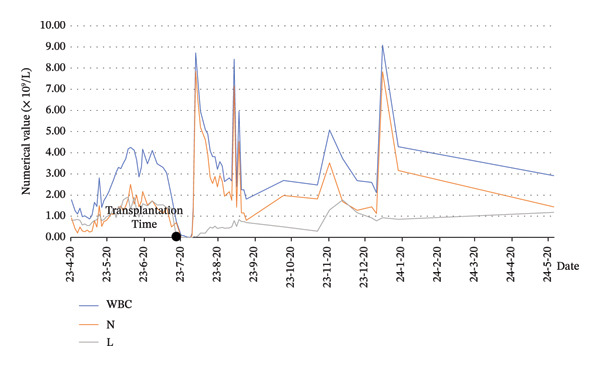
Changes in patients’ white blood cells before and after transplantation.

**Figure 3:**
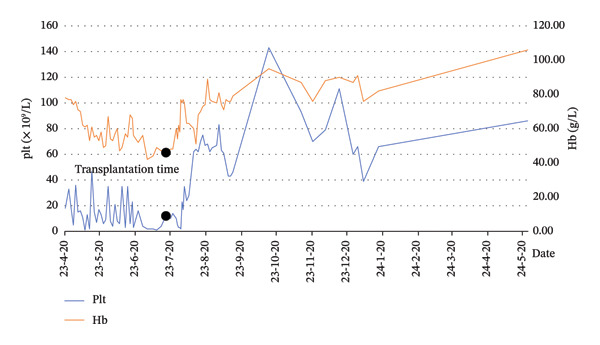
Changes in platelets and hemoglobin before and after transplantation in patients.

The patient was admitted to our hospital for further treatment. On admission (April 20, 2023), blood tests revealed the following: white blood cell count 1.78 × 10^9^/L, absolute neutrophil count 0.81 × 10^9^/L, red blood cell count 2.46 × 10^9^/L, hemoglobin 78 g/L, platelet count 18 × 10^9^/L, and reticulocyte percentage 0.55%. Bone marrow pathology showed low proliferation (approximately 20%) with visible granulocytic and erythroid lineage cells, but rare megakaryocytes, as shown in Figure 1. Flow cytometry analysis revealed no evidence of PNH‐clonal expression in either red blood cells or white blood cells. Myeloid hematological tumor gene mutation test detected no pathogenic mutations. The patient’s conventional karyotyping was 46, XX. Initial treatment consisted of high‐dose immunoglobulin, a 3‐month course of immunosuppressants, and TPO agonists, along with a total of 15.5 units of red blood cells and 25.0 therapeutic units of platelets; however, hematopoietic function did not recover. Follow‐up bone marrow examination showed minimal hematopoietic cells. Pretransplant assessment of the patient’s breast cancer: I. Tumor characteristics: (1) Stage and grade: pT2N0M0, Stage IIA, Luminal B type, HER2‐positive. (2) Activity: Tumor markers CA125 and CA153 remained within normal ranges, consistent with inactive disease. (3) The patient has previously undergone multiple courses of chemotherapy and targeted therapy. II. Organ function reserve: (1) Bone marrow examination shows no evidence of metastatic cancer. (2) No significant abnormalities were observed in cardiac, pulmonary, hepatic, or renal function. Transplantation matching revealed only one haploidentical sibling donor (sister, 6/12 match in GVH direction). The patient (height 156 cm, weight 43 kg, and body surface area 1.47 m^2^) received a conditioning regimen comprising busulfan (BU) (3.2 mg/kg/d for 1 day), fludarabine (Flu) (25 mg/m^2^/d for 5 days), cyclophosphamide (50 mg/kg/d for 2 days), and antithymocyte globulin (2.5 mg/kg/d for 4 days). Preventive measures include the use of phenytoin to treat seizures caused by BU, the use of mesna to treat cyclophosphamide‐induced hemorrhagic cystitis, and the combination of mycophenolate mofetil and cyclosporine A to prevent graft‐versus‐host disease (GVHD). PEG‐G‐CSF and thrombopoietin were administered to assist hematopoietic reconstruction. Letermovir and cotrimoxazole were used to prevent CMV and *Pneumocystis carinii* infections, respectively. On Day 7 posttransplantation (July 28, 2023), the patient developed fever, cough, diarrhea, and multiple oral ulcers. Biapenem, acyclovir, and posaconazole were added to control infection. Neutrophil engraftment was achieved on Day 10 (July 31, 2023) (Figure 2), and platelet engraftment on Day 15 (August 5, 2023) (Figure 3). During hematopoietic reconstruction, the patient experienced fever. Blood NGS detected human herpesvirus 6B. Treatment included reducing cyclosporine A dosage and administering human immunoglobulin with foscarnet. On Day 20 posttransplantation (August 10, 2023), the patient developed a generalized itchy rash, indicative of acute GVHD (aGVHD). Treatment included methylprednisolone and umbilical cord blood mesenchymal stem cell infusion, which gradually controlled both infection and rejection. Day 30 posttransplantation (August 20, 2023), blood tests showed recovery: white blood cell count 3.58 × 10^9^/L, hemoglobin 89 g/L, and platelet count 68 × 10^9^/L. Bone marrow examination indicated active hematopoiesis, and STR testing showed 99.30% donor chimerism. Cutaneous GVHD (mainly manifested as generalized pruritus, with scattered maculopapular rashes accompanied by pain on both hands, feet, forearms, and lower legs) has improved significantly, and no other manifestations of rejection were observed.

The patient was discharged on September 12, 2023, with weekly outpatient follow‐ups. On Day 90 posttransplantation, the white blood cell count was 2.69 × 10^9^/L, necessitating PEG‐G‐CSF and immunoglobulin administration. Day 120 follow‐up bone marrow examination showed stable disease with 97.81% donor chimerism. The white blood cell count was 2.48 × 10^9^/L, requiring another PEG‐G‐CSF dose. The patient continues to receive antirejection therapy, viral prophylaxis, and immune enhancement treatment.

## 3. Discussion

Approximately 66% of newly diagnosed breast cancer patients require chemotherapy. Historically, neoadjuvant chemotherapy for breast cancer primarily involved anthracycline‐based regimens, which were limited by severe gastrointestinal side effects and cumulative cardiotoxicity. Currently, paclitaxel has emerged as the first‐line agent in breast cancer chemotherapy, offering improved tolerability and efficacy [3]. In our case, the patient received neoadjuvant therapy consisting of six cycles of TCbHP (albumin‐bound paclitaxel, carboplatin, pertuzumab, and trastuzumab), followed by seven cycles of targeted therapy (pertuzumab and trastuzumab). Subsequently, she developed Grade IV myelosuppression that persisted for 3 months despite various hematopoietic stimulation therapies. This led to a diagnosis of severe AA (SAA).

The incidence of chemotherapy‐associated myelosuppression in breast cancer patients is 67.16%, and the incidence of severe myelosuppression is 51%, and secondary infections, bleeding, and even drug‐induced AA may occur, which may lead to interruption of the complete cycle of chemotherapy or inability to carry out the chemotherapy or even endanger the life [4]. Myelosuppression occurs by two main mechanisms: destruction of hematopoietic stem cells and damage to the hematopoietic microenvironment [5]. Chemotherapeutic drugs mainly have a killing effect on proliferating cells, while hematopoietic stem cells and tumor cells both have a strong proliferation ability; therefore, chemotherapeutic drugs kill tumor cells and also cause damage to hematopoietic stem cells. The hematopoietic microenvironment is the site of proliferation and differentiation of hematopoietic stem cells and maintains the hematopoietic homeostasis of the body by releasing various cytokines and chemokines [6]. However, repeated chemotherapy may cause alterations in the microenvironment, mainly including damage to bone marrow stromal cells and damage to the bone marrow sympathetic nervous system, which causes long‐term damage to the body’s hematopoiesis [7]. Chemotherapy‐induced SAA is a rare but potentially life‐threatening complication in breast cancer treatment, but its pathogenesis has not been fully clarified, and its incidence has not been reported in the literature.

Management of chemotherapy‐induced SAA presents a unique challenge, as it requires balancing the treatment of the underlying malignancy with the need to restore hematopoiesis.

Although the pathogenesis of secondary SAA has not been fully elucidated, it is believed that the occurrence of SAA is related to the significant reduction of hematopoietic stem cells and progenitor cells caused by hyperactivity of T lymphocyte immune function. In this case, after chemotherapy, the patient’s examination results indicated a decrease in all three blood cell lines and low bone marrow hyperplasia. Despite treatments such as hematopoietic promotion and blood cell transfusion, the patient’s blood picture did not improve significantly. Moreover, the lymphocyte subset analysis suggested the presence of active T lymphocytes. Given that the patient had no HLA‐matched sibling donor and was over 40 years old, cyclosporine was administered for IST. However, there was no significant improvement in the blood picture after treatment, and the patient experienced nausea and anorexia after taking cyclosporine orally. Therefore, tacrolimus was substituted and combined with intravenous immunoglobulin pulse therapy. The patient’s granulocyte count rose somewhat, but the low platelet count and anemia did not improve. Statistically, approximately 60% of AA patients require further treatment after frontline standard IST [8]. Further optimal treatment options have not been well‐established by numerous studies, often requiring comprehensive consideration based on factors such as the patient’s age, drug sensitivity, availability of a matched donor, and so forth. In this particular case, the patient is a middle‐aged female who, after 3 months of various hematopoietic‐stimulating treatments, showed signs of bone marrow failure upon reevaluation. In addition, there was a half‐matched donor available. Therefore, we ultimately chose to proceed with haplo‐HSCT as the treatment.

In HSCT, the first consideration in donor selection is HLA‐matched allogeneic compatibility [9], and MSD‐HSCT has resulted in a 5‐year overall survival rate of greater than 80% for patients with SAA [10], but the probability of having a sibling with compatible HLA‐matches is very low, and the probability of finding an allogeneic nonconsanguineous donor is also low, so the selection of haplo‐HSCT provides an alternative to the patients with SAA. Xu et al. compared the outcomes of 89 patients with SAA who underwent first‐line haplo‐HSCT with 69 patients with SAA who underwent concurrent MSD‐HSCT, and found that, although the haploidentical transplantation group had a higher incidence of aGVHD of II–IV and chronic GVHD, there was no statistically significant difference in the time to neutrophil engraftment and overall survival at 3 years between the two groups, compared to the MSD‐HSCT group [11]. Im et al. used a haploidentical transplantation model with ex vivo T‐cell depletion to treat 12 cases of immature SAA and obtained good implantation with a low incidence and mild acute and chronic GVHD and no GVHD‐related deaths [12].

The choice of pretreatment regimen is a key component of haplo‐HSCT. The objectives are (1) to eliminate abnormal cells or tumor cells in the patient’s body to minimize recurrence and (2) to inhibit or remove the patient’s immune system to provide conditions for the implantation of hematopoietic stem cells and to prevent rejection of the grafts. For patient types with relatively high implantation failure rates such as patients ≥ 40 years old, long disease duration, and heavily transfused patients, an intensive pretreatment regimen was given to ensure implantation by adding Flu or BU or a small‐dose TBI on top of CY‐ATG [13]. The “Beijing protocol” (BU/CY + ATG), which was successfully implemented by a team led by Prof. Huang Xiaojun in China, was based on intensive immunosuppression with the addition of BU for myeloablative conditioning [14]. BU is an alkylating agent that disrupts the structure and function of DNA by alkylating with guanines within cellular DNA, but it has a relatively narrow therapeutic index, with low drug exposures associated with an increased risk of graft failure and disease recurrence, and high drug exposures associated with an increased incidence of hepatic complications, particularly veno‐occlusive disease (VOD). There is no consensus among transplant centers on the dosage of BU for SAA, but at our center, allogeneic HSCT for SAA is usually performed with BU 3.2 mg/(kg·d) 1 d (−4 d) and the addition of prostaglandin to improve microcirculation and prevent VOD. Studies have shown that the Beijing protocol not only has a higher implantation rate but also a higher incidence of GVHD. In our center, haplo‐HSCT is routinely performed with the addition of Flu [15], which mainly removes recipient lymphocytes to ensure complete implantation of donor stem cells and also reduces GVHD. The current patient outcome suggests that this transplantation regimen is effective, with fewer associated complications.

We searched the relevant literature databases and did not find any reports related to haplo‐HSCT for AA after chemotherapy for breast cancer. Successful transplantation in this case reduces the risk of prolonged thrombocytopenia due to bone marrow suppression after chemotherapy for the patient’s solid tumors, the need to rely on transfusion of blood cells, and hemorrhage, but a long period of follow‐up continues to be needed.

## 4. Conclusion

The middle‐aged female patient, after being diagnosed with breast cancer, underwent multiple neoadjuvant therapies. Subsequently, she developed pancytopenia and was diagnosed with SAA through bone marrow aspiration and other examinations. After 3 months of IST, her hematopoiesis did not recover. In the absence of a suitable fully HLA‐matched donor, her sister was chosen as the haploidentical donor for haplo‐HSCT. Haplo‐HSCT involves a high degree of HLA mismatch, which carries a high risk of graft rejection, a relatively high incidence of GVHD, and susceptibility to severe infections. Moreover, the patient’s prior chemotherapy might have caused long‐term damage to the bone marrow, potentially affecting the transplant outcome. This case successfully utilized haplo‐HSCT to treat SAA following breast cancer chemotherapy, providing a therapeutic option for similar patients. It also aids in considering the risks and benefits of haplo‐HSCT in populations with an increased risk of hematological diseases after chemotherapy for solid tumors. However, it is crucial to be mindful of the risks and challenges associated with haplo‐HSCT and to strengthen management and monitoring before and after transplantation to improve success rates and patient prognosis. With the continuous development and refinement of haplo‐HSCT technology, its application in the treatment of diseases such as SAA is expected to broaden, bringing benefits to more patients.

## Funding

No funding was received for this study.

## Conflicts of Interest

The authors declare no conflicts of interest.

## Data Availability

The data that support the findings of this study are available from the corresponding author upon reasonable request.
